# An Integrated Deep Learning Method towards Fault Diagnosis of Hydraulic Axial Piston Pump

**DOI:** 10.3390/s20226576

**Published:** 2020-11-18

**Authors:** Shengnan Tang, Shouqi Yuan, Yong Zhu, Guangpeng Li

**Affiliations:** 1 National Research Center of Pumps, Jiangsu University, Zhenjiang 212013, China; 2111811013@stmail.ujs.edu.cn (S.T.); zhuyong@ujs.edu.cn (Y.Z.); 2221911015@stmail.ujs.edu.cn (G.L.); 2 State Key Laboratory of Fluid Power and Mechatronic Systems, Zhejiang University, Hangzhou 310027, China; 3 Ningbo Academy of Product and Food Quality Inspection, Ningbo 315048, China

**Keywords:** convolutional neural network, continuous wavelet transform, intelligent fault diagnosis, hydraulic axial piston pump

## Abstract

A hydraulic axial piston pump is the essential component of a hydraulic transmission system and plays a key role in modern industry. Considering varying working conditions and the implicity of frequent faults, it is difficult to accurately monitor the machinery faults in the actual operating process by using current fault diagnosis methods. Hence, it is urgent and significant to investigate effective and precise fault diagnosis approaches for pumps. Owing to the advantages of intelligent fault diagnosis methods in big data processing, methods based on deep learning have accomplished admirable performance for fault diagnosis of rotating machinery. The prevailing convolutional neural network (CNN) displays desirable automatic learning ability. Therefore, an integrated intelligent fault diagnosis method is proposed based on CNN and continuous wavelet transform (CWT), combining the feature extraction and classification. Firstly, CWT is used to convert the raw vibration signals into time-frequency representations and achieve the extraction of image features. Secondly, a new framework of deep CNN is established via designing the convolutional layers and sub-sampling layers. The learning process and results are visualized by t-distributed stochastic neighbor embedding (t-SNE). The results of the experiment present a higher classification accuracy compared with other models. It is demonstrated that the proposed approach is effective and stable for fault diagnosis of a hydraulic axial piston pump.

## 1. Introduction

Owing to the advantages of fast response, high power density and high stability, hydraulic transmission systems play a critical role in industry [1,2,3]. The hydraulic axial piston pump is considered the critical power source of the hydraulic transmission system, and it is meaningful to ensure its stable operation. On account of the severe conditions of high temperature, high pressure and heavy working load, the incident and unexpected faults may lead to enormous economic losses and potential safety impacts [4,5,6]. Therefore, it is significant and valuable to exploit the effective and accurate fault diagnosis methods for the stability and reliability of the system.

In light of fault diagnosis in hydraulic axial piston pumps, numerous studies have been emphasized conventional methods [7,8]. Traditional fault diagnosis methods are mainly based on the analysis of the mechanism, characteristic frequency or the extraction of fault feature. In consideration of the fuzzy fault characteristics and complex structure of the pump, it is difficult to use traditional subjective manual diagnosis methods to exactly achieve its fault diagnosis.

With the development of artificial intelligence, intelligent fault diagnosis technologies have aroused increasing attention of researchers [9,10,11]. Intelligent diagnosis methods present the powerful processing capability for mechanical big data, which no longer rely on professional knowledge and diagnostic experience, as was previously the case. It is worth pointing out that machine learning based diagnostic methods are viewed as the typical representative. Amar et al. constructed a neural network model based on a vibration spectrum for bearing fault diagnosis [12]. As one of classical and popular machine learning methods, support vector machine (SVM) has been employed to achieve the non-linear classification [13,14,15]. Moreover, SVM based methods were employed to investigate the fault diagnosis of rotating machinery [16,17]. Contrastive analysis was performed by Han et al., primarily on random forest, annual neural networks, and SVM for fault diagnosis in rotating machinery [18].

Due to the limitations of traditional machine learning in feature extraction and model training, deep learning (DL) based technology motivates the investigation of intelligent fault diagnosis [19,20,21]. As one of the effective and precise DL methods, convolutional neural network (CNN) has been regarded as a potential tool for machinery fault diagnosis. The characteristics of weight sharing and down-sampling means that CNN outperforms other deep network models. To analyze the fault type and severity, a LiftingNet framework was developed, and the varying rotating speed and operation environment were taken into account [22]. Liang et al. used wavelet transform to accomplish the transformation of signal into images, and established CNN for fault classification [23]. By introducing a simplified shallow information fusion, a new CNN model was employed for fault diagnosis towards the high-speed train axle-box bearing [24]. The training time of the network was effectively reduced and the diagnostic performance was promoted in the meantime. By combining Hilbert–Huang transform (HHT) and CNN, Gao et al. developed a novel approach for fault diagnosis of micro-electromechanical system inertial sensors [25]. The proposed method presents remarkable performance in comparison to modern methods. In order to figure out the imbalanced distribution of machinery data, Lei et al. carried out a deep normalized CNN for bearing fault diagnosis [26]. Based on LeNet-5, new diagnostic methods were developed for the fault diagnosis of pumps, the ability of CNN in image processing was admirably demonstrated in the form of predicted fault classification results [21,27]. By integrating CNN and recurrent neural network, Shenfield et al. constructed a novel deep model for the fault detection and distinction of bearing [28]. Motivated by transfer learning, a new method called deep convolution domain-adversarial transfer learning was developed for fault diagnosis of rolling bearings [29]. Based on the transfer learning model of ResNet-50, a multi-scale deep model was constructed for bearing fault diagnosis, enhancing the robustness and generalizability of the model [30]. Moreover, the proposed model was compared with many other models, SVM, CNN, CNN with maximum mean discrepancy (MMD) and so on. The advantage of the established model was fully proved. Inspired by the analysis of the single signals, Ye et al. constructed a new model based on deep neural network, employing the feature fusion on the signals from multi-channel sensors [31]. In contrast with other intelligent methods with signals from a single sensor, including the Back-Propagation neural network and SVM, the proposed method was demonstrated to be more effective and accurate for fault diagnosis. Using continuous wavelet transform (CWT) as a preprocessing method, a CNN was employed for bearing fault diagnosis [32]. To solve the problem of insufficient fault data, a combined intelligent approach was developed based on CNN, nonlinear auto-regression neural network and CWT. The performance was verified by two imbalanced datasets, including bearing and gear [33]. By integrating CNN and an extreme learning machine, Chen et al. developed a novel method for fault diagnosis of gearboxes and motor bearings, using CWT for the conversion of raw signal [34]. As a prevailing dimension reduction algorithm in machine learning, t-distributed stochastic neighbor embedding (t-SNE) has been employed for the visualization of the feature learned by the CNN model [35,36,37]. Although many studies based on DL methods have achieved some successful results for fault diagnosis of bearing and gearing, the research on pumps are still insufficient, especially for hydraulic axial piston pumps. Furthermore, owing to the complex structure and the difficulty in acquiring the fault data, it is a great challenge to accomplish the precise fault diagnosis of a hydraulic axial piston pump.

In this paper, three key contributions are made in the following:

(1) Known as one of the most widely-used rotating machinery in many fields, fault diagnosis of hydraulic axial piston pumps is considered to be necessary and significant in engineering applications. Moreover, the present intelligent fault diagnosis methods are mainly focused on the bearing, gearing and gearbox, the research on hydraulic axial piston pumps is lacking.
(2) In consideration of the superiority of wavelet transform in nonlinear signal processing, CWT is integrated into the approach to achieve the transformation of the time-frequency representations from raw vibration signals.
(3) The limitations of traditional diagnostic methods and common intelligent fault diagnosis approaches are effectively overcome, the proposed diagnosis method will provide an important concept for exploring the new diagnostic methods.

Therefore, this research puts emphasis on the intelligent fault diagnosis methods of the hydraulic axial piston pump. Firstly, basic theory of CNN is briefly introduced in Section 2. In Section 3, in order to reduce the difficulty of feature extraction, CWT is selected for preprocessing of raw vibration signals. In light of the superiority of CNN in the feature learning, a new CNN model is employed for fault diagnosis of the pump. In Section 4, the diagnostic performance of the proposed method is validated by the experiments, and the effectiveness of the model is displayed by confusion matrix and t-SNE. Furthermore, the comparisons are performed with different CNN based models.

## 2. Basic Algorithm Theory

### 2.1. Brief Introduction to Convolutional Neural Network

In light of the diverse fault classification methods and the nonlinear characteristics of machinery big data, deep learning based technology aroused the concern of researchers in the fault diagnosis field [38,39,40]. As one of the prevailing and effective representatives, CNN presents a powerful automatic learning capability for useful and distinguished features, compensating for the deficiencies of the fully connected feedforward neural network in multiple parameters and local invariance.

Generally, typical CNN structure is composed of different layers, involving a data input layer, convolution layer, ReLU (Rectified Linear Unit) layer called the activation layer, a pooling layer and a fully connected layer. The structural layers can be used to complete the feature extraction and final classification. CNN shows superiority over other DNN methods owing to three main traits: involving local connection, weights sharing and down-sampling. Therefore, a reduction in network parameters needing to be optimized can be achieved, and the bottleneck of overfitting can be resolved to a certain extent during feature learning [41,42].

Compared to the structures of other deep learning models, convolution layer and subsampling layer are distinct for the CNN models. In terms of the local receptive field in the convolutional layer, the size is the same as the convolutional kernels. Convolutional kernel is also named filters and is considered to be a local window. In a local window, two layers of adjacent neurons are connected to each other [43,44]. 

Convolutional kernel can be viewed as a linear time-invariant system, the feature map of the next layer can be calculated by:

(1) $$x_{j}^{l}=f(\sum_{i\in M_{j}}x_{i}^{l-1}\times k_{j}^{l}+b_{j}^{l})$$

where, (×) denotes the operation of the convolution. x represents the input of the network. $k_{j}^{}$ denotes convolutional kernel. Then, the convolution of the kernel is performed on the input data. $b_{}^{}$ is the bias and is introduced during the process. Ultimately, the activation function f could be employed for obtaining output nonlinear features. 

The pooling layer is also called the sub-sampling layer, and can further reduce the number of parameters on the basis of local connection. Furthermore, it can enhance the generalization ability of model.

The process of the pooling operation can be expressed by,

(2) $$a_{j-s}^{l}=f(w_{j}^{l}down(M_{j}^{l-1})+b_{j}^{l})$$

Among them, down(⋅) means the calculation of the maximum or the mean values in regard to the convolved features. In the function, f, $M_{j}$, $w_{j}^{l}$, and $b_{j}^{l}$ represent the feature map, weight and the bias, respectively.

As for the fully connected layer, a softmax regression model can be considered as effective and accurate in conducting multiclass classification.

### 2.2. Basic Principle of Continuous Wavelet Transform

With regard to the basic theory of WT, the relative studies can provide some references [45]. The mother wavelet can be presented as follows,

(3) $$\psi_{u,s}(t)=\frac{1}{\sqrt{s}}\psi (\frac{t-u}{s})$$

Among them, $\psi_{u,s}$ represents the wavelet dictionary, which is generated by a single wavelet; s and u are two variables, respectively; the parameter s denotes the scale; u denotes the translation and u∈R, which is employed to control the translation of the wavelet function.

The WT can be accordingly calculated by,

(4) $$W_{f}(u,s)=\langle f,\psi_{u,s}\rangle =\frac{1}{\sqrt{s}}\int x\left(t\right)\psi (\frac{t-u}{s})dt$$

Owing to the advantages in processing nonstationary signal, CWT was carried out to accomplish the image transformation for the fault data of the hydraulic axial piston pump in each condition. Compared with regular wavelets, ComplexMorlet presents good resolution in the time-domain and frequency-domain. Hence, ComplexMorlet is selected as the wavelet basis function.

## 3. Proposed Intelligent Fault Diagnosis Method

### 3.1. Data Description

**Figure 1:**
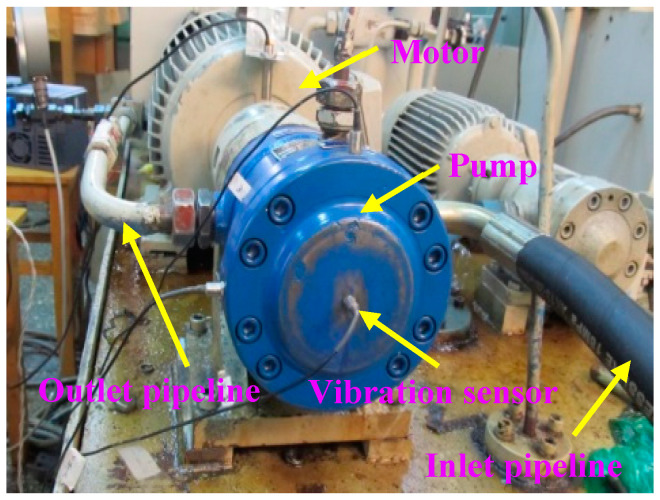
The test system for fault experiment.

The experiments were performed on a hydraulic axial piston pump test platform, as shown in Figure 1. The test bench was primarily composed of a motor, a pump and an acceleration sensor et al. The object of this test was a swash-plate axial piston pump with seven plungers. The rated speed of the pump was 1470 r/min, which means the corresponding rotary frequency was 24.5 Hz. In regard to the data acquisition equipment, a multi-function data acquisition card is provided by National Instruments (NI) Company (Austin, TX, USA). The model number of the equipment is USB-6221. The fault vibration signals are acquired from the data acquisition system. As for each condition, the sampling frequency was set to 10 kHz.

During the experiments, five different health conditions were simulated, mainly including normal and faulty states. The obtained data were employed for the following fault diagnosis to demonstrate the classification of the CNN model. The index names represent the index corresponding to the name of the fault category. Specifically, it is a processing step on the input data before feeding it to the neural network. The specific descriptions of the five conditions are expressed in Table 1.

### 3.2. Data Preprocessing

In common fault diagnosis methods, data preprocessing technologies are usually used to achieve feature extraction by complex steps [46]. Combining signal acquisition, feature extraction and fault classification, intelligent techniques could be considered as a potent direction in developing novel fault classification methods [47]. However, the requirements for data input should be eligible for the training of deep network models, especially, image/graph inputs are requested for methods such as 2D CNN.

In addition to CWT, there are many other processing methods for transforming the signals into images, including short time Fourier transform (STFT), S-transform (ST), discrete wavelet transformation (DWT) and cyclic spectral coherence (CSCoh). STFT uses a fixed window function and is usually used to analyze piecewise stationary signals or quasi-stationary signals. However, the frequency and time resolution cannot be taken into account in the meantime [48]. DWT is a discretization to the scale and translation of basic wavelet and generally refers to two-scale wavelet transform. Compared with CWT, DWT resolves the problem of calculated quantity [49]. As the inheritance and development of WT and STFT, ST eliminates the selection of window function and enhances the deficiency of fixed window width. Moreover, the features extracted by ST are not sensitive to noise [50]. Compared to conventional cyclostationary analysis, CSCoh can effectively overcome noise interference and obtain the potential fault information via the analysis of the relationship between the spectral frequency and cyclic frequency [51].

**Figure 2:**
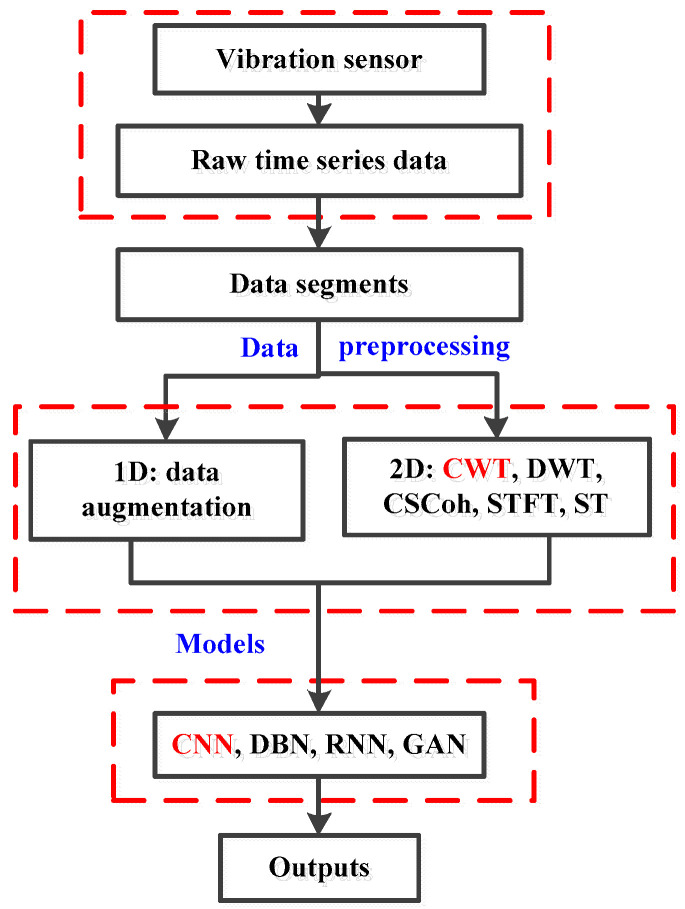
Flowchart of fault data preprocessing. CWT: continuous wavelet transform; DWT: discrete wavelet transformation; CSCoh: cyclic spectral coherence; STFT: short time Fourier transform; ST: S-transform; CNN: convolutional neural network; DBN: deep belief networks; RNN: recurrent neural networks; GAN: generative adversarial networks.

From Figure 2, with regard to each fault type, the acquired raw time series are firstly divided into various data segments. Each segment involves 1024 sampling points. Then on account of the input requirements of diverse models, different preprocessing methods can be employed to the following steps. The amount of one dimensional (1D) data can be increased by data augmentation for expending the training datasets. As for the models of two dimensional (2D) input, the segments should be converted into 2D images or matrixes through time-frequency analysis methods, including STFT, ST, CWT and CSCoh [52,53,54,55]. Furthermore, the obtained 2D images are taken as the input of the established CNN model. Deep models can include CNN, deep belief networks (DBN), recurrent neural networks (RNN), and generative adversarial networks (GAN). CNN is selected in this work. Through the training and testing of the network, the outputs present the performance of the model, including the training loss and the classification accuracy.

### 3.3. Proposed Intelligent Method

In view of the excellent performance of the popular CNN model in image identification and classification, a new intelligent method based on the CNN model is proposed for fault diagnosis of the hydraulic pump. Firstly, the vibration signal is acquired as raw data. Secondly, the vibration signals are transformed into time-frequency images for the establishment of training and testing datasets. Then, a CNN model is constructed and trained with the training datasets obtained above. Finally, the testing datasets are employed to test and validate the classification performance. Hence, the intelligent fault diagnosis is accomplished for hydraulic axial piston pump.

**Figure 3:**
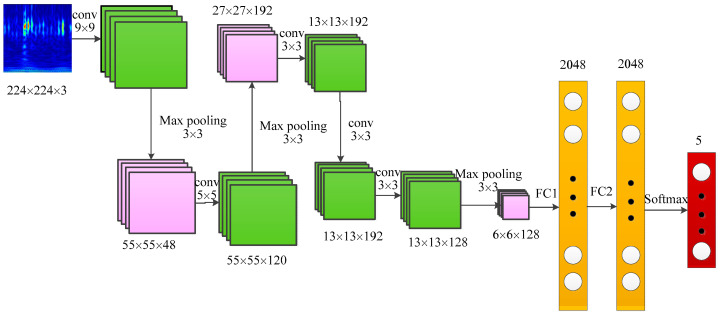
The framwork of the proposed CNN model.

It is composed of five varying convolutional layers (Conv), three sub-sampling layers and three fully-connected layers (Figure 3).

Maxpooling is used to reduce the dimension of features and overfitting of the model. The size of the pooling area is 3 × 3 for each pooling layer. In order to inhibit overfitting and gradient vanishing of the model, the operation of dropout is taken into account. Namely, the dropout layer is introduced during the fully connected layers. Owing to the five different fault types for hydraulic axial piston pump, the output of the network is set as five. During the classification step, the Softmax function is employed to convert the prediction results of the model into the exponential function, to ensure the non-negative probability. Moreover, it can guarantee that the sum of the probabilities of each prediction is equal to one.

In order to obtain the optimized structural parameters of CNN, the gradient descent algorithm can be employed. It can be understood that the parameters of the network will be updated according to the gradient information from the back-propagation. Then the value of the cross-entropy loss function will be reduced and finally, the learning of the network will be accelerated. Adam is a typical and effective optimization algorithm proposed by Kingma and Ba [56]. Adam integrates Momentum with the RMSprop algorithm, adopting Momentum and an adaptive learning rate to accelerate the convergence speed. Moreover, Adam presents superiority in processing non-stationary objectives and problems with noisy and/or sparse gradients.

## 4. Validation of Proposed CNN Model

### 4.1. Input Data Description

In the operation process of equipment, mechanical faults may lead to various signals, including impact, environmental noise and other features. It is of great difficulty to classify diverse fault types only from 1D time-domain/frequency-domain analyses. Therefore, 2D time-frequency analysis is considered to be more effective for processing nonlinear signals.

**Figure 4:**
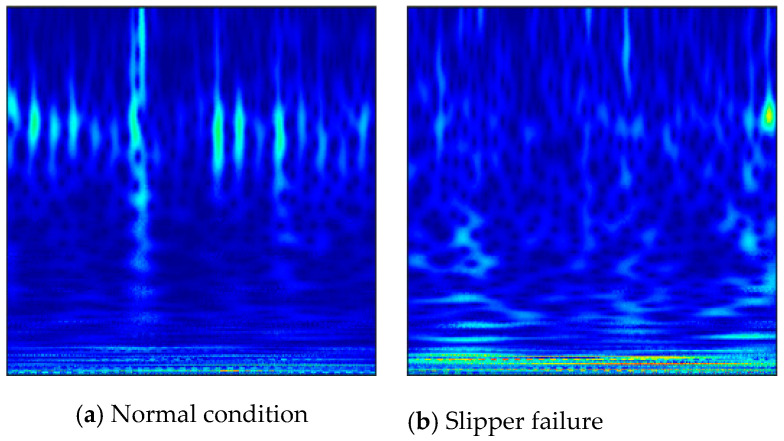

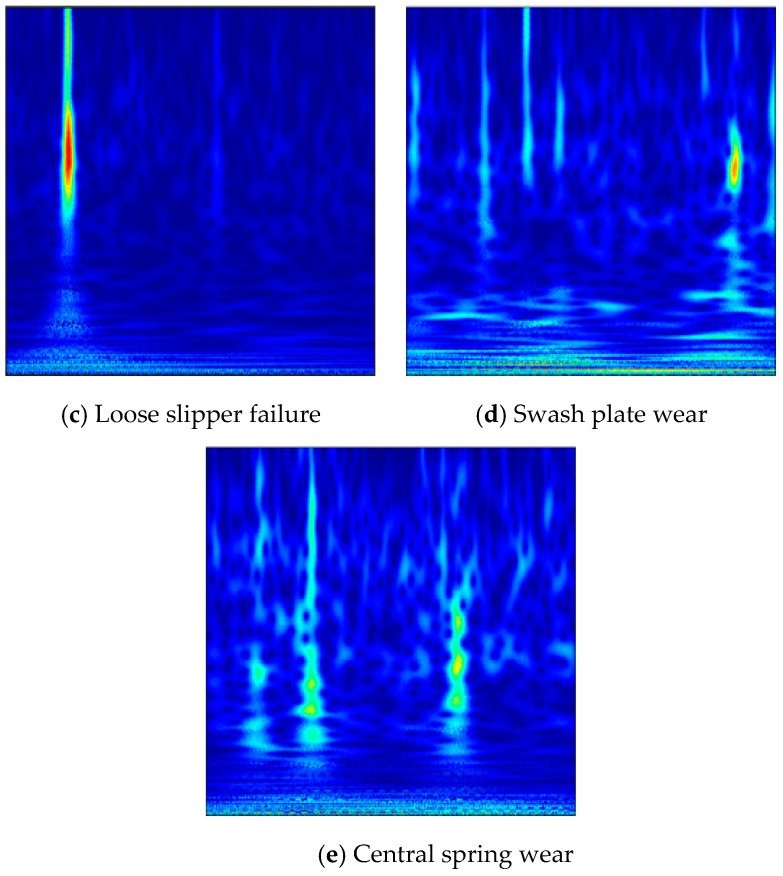
Time-frequency image representations under 5 conditions.

CWT is selected as the preprocessing method for this research. During the CWT operation, ComplexMorlet is chosen as the wavelet basis function. The bandwidth and center frequency are both three, and the length of the scale sequence used in wavelet transform is 256. The time-frequency images of five conditions are displayed in Figure 4. The images of different fault types are similar to a certain degree. It can be found that there was some distinction in changing fault types. The frequency varied with time as depicted in representations under various states. However, it is hard to distinguish various fault types based on experience and diagnostic knowledge. It just provides sufficient space for automatic feature learning of the following established deep CNN model. It could demonstrate the mining capability of the implied characteristics from such similar representations.

In the obtained samples, there was a total of 6000 time-frequency images, and each type of fault involved 1200 images. Before inputting into the CNN model, the strategies of data transform were used for the adjustment of raw image size. The image size was transformed into the same size of 224 × 224. The random horizontal flip was carried out on the samples in the training dataset. The samples were randomly divided into a training dataset and testing dataset in the ratio of 7:3. Namely, there were 840 training samples and 360 testing samples under each fault category, respectively. The detailed data are described in Table 2. Furthermore, in order to validate the diagnosis performance of the model and effectively avoid overfitting, only the training samples were used for updating the weights and bias of the model in the training process. The network model has never been exposed to the test samples.

### 4.2. Parameter Selection for the Proposed Model

In consideration of the great influence on the classification performance, some critical parameters were analyzed and discussed, including epoch, batch size, and the number and size of the convolutional kernel. The suitable network model will be established via the optimization of the parameters above. 

Small epochs will result in the undesired effect of fitting towards the model. If a big epoch is selected, the classification accuracy may be enhanced, but it will bring about a higher time cost. Therefore, it is vital to choose an appropriate epoch for the construction of the model.

**Figure 5:**
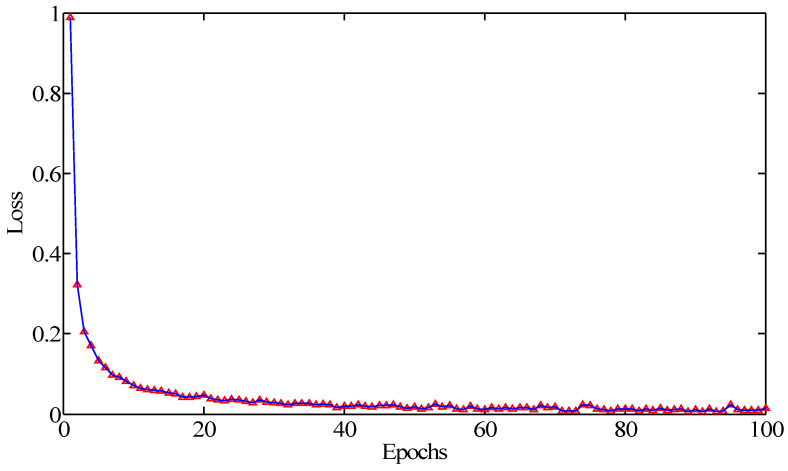
The tendency of the relationship between the training loss and epoch.

**Figure 6:**
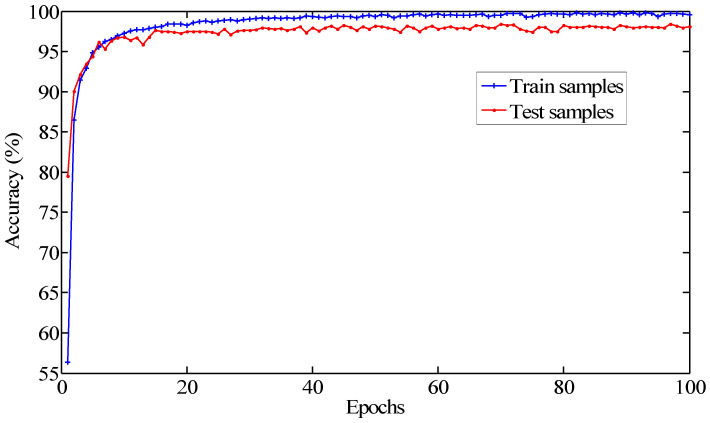
The tendency of the relationship between the classification accuracy and epoch.

In order to study the convergence process of the network model, we set the epoch as 100 and repeated the trials 10 times. The average results were recorded as the final diagnostic accuracy. As depicted in Figure 5 and Figure 6, the initial training loss was more than one. It gradually decreased with the increase in the epoch. At the beginning, the classification accuracy was lower than 80%. It increased gradually with the increase in the epoch contrary to the training loss. When the epoch was over 15, the testing accuracy was more than 94%. When the epoch was more than 30, the testing accuracy reached over 96%. With the further increases in training epochs, the loss value tended to be a small value and remained stable. Meanwhile, the classification accuracy slightly fluctuated, indicating that the CNN model has been trained to converge. Hence, the training epoch was chosen as 30 in the following research studies.

Large batch size may lead to a faster convergence speed of the network model. Then, the training time can be reduced, and the training curve of the model will be smoother, which can improve the stability of the model. However, with the increase in batch size, the number of adjustment weights and offset will be reduced, and the performance of the model will be reduced, resulting in a reduction in the generalization ability of the model. The smaller batch size is favorable for improving the effect of classification, but it will bring about a higher computation cost. If the batch size is smaller than the number of categories in the datasets, the model will not converge. Therefore, proper batch size is necessary for the selection of the parameters of the model.

In this research, the batch size was selected in the light of multiple factors, involving the sensitivity of graphics processing unit (GPU) to the value of 2^n^, eight multiples. The batch size is divisible by the total number of training samples. In consideration of computational time and classification accuracy, the batch was chosen as 56.

**Figure 7:**
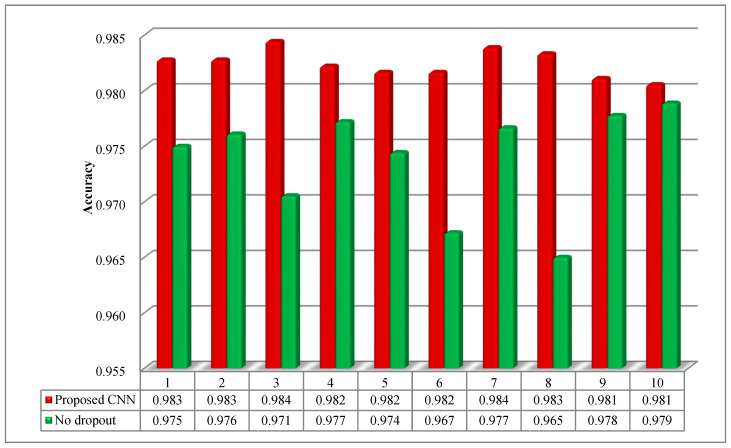
The testing accuracy with and without dropout layers for 10 trials.

To inhibit overfitting of the model, the operation of dropout was conducted among the FC layers. The models with and without dropout layers were investigated to explore the effect on the classification accuracy. As can be seen from Figure 7, the model without dropout layers presents remarkable fluctuation in 10 trials. Moreover, the accuracy is less than that of the proposed CNN model. It can be demonstrated that the proposed CNN model is stable, and the design of dropout layers enhances the performance of the model.

**Figure 8:**
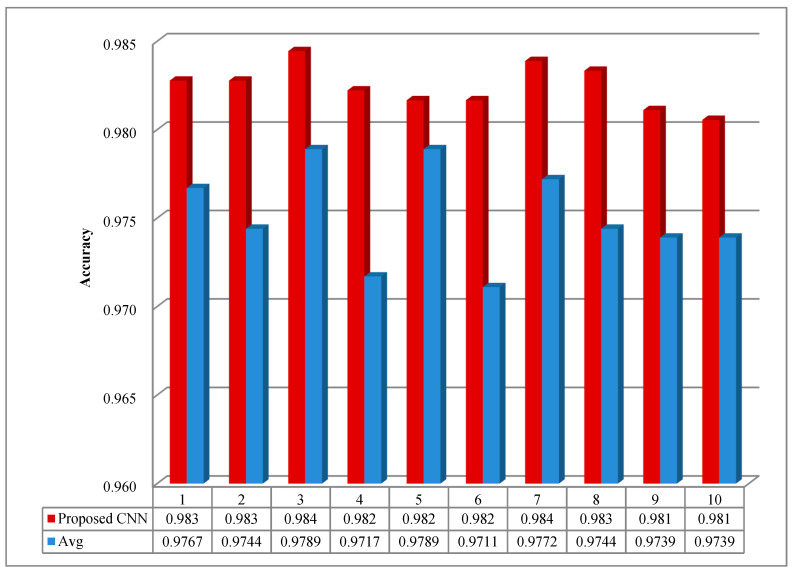
The testing accuracy with average pooling and maxpooling.

In order to probe the influence of the pooling layer on the classification performance of the CNN model, the average pooling was employed for comparisons. As shown in Figure 8, the classification accuracy of the model with average pooling is lower than 98%, which is inferior to that with the maxpooling operation. Therefore, the operation of maxpooling was selected to achieve the reduction in the dimension of the data.

### 4.3. Performance Validation of the Proposed Model

To validate the reliability and stability of the proposed model, 10 repeated trials were conducted through adopting the optimized training and structural parameters. The maximum pooling method was employed to the dimension reduction in features to be learned. Adam optimizer was used for the optimization of the model, and the original learning rate was set as 0.0002. 

**Figure 9:**
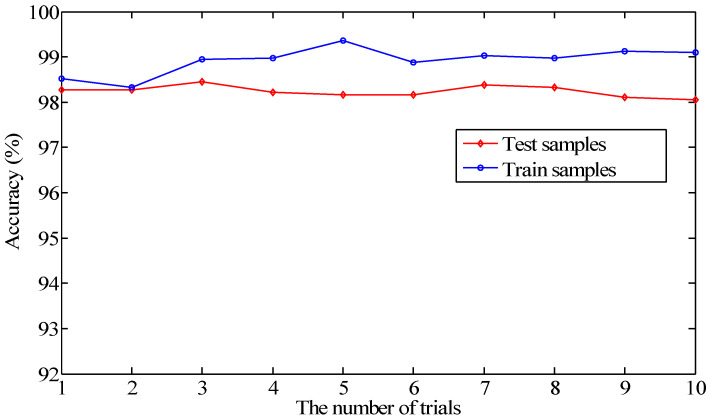
The accuracy curve of the training and testing samples in the proposed method.

As shown in Figure 9, the difference between testing and training accuracy is not very obvious, and the average testing accuracy of the 10 trials all exceeded 98%. Therefore, the effectiveness of the parameters can be demonstrated for the proposed model.

**Figure 10:**
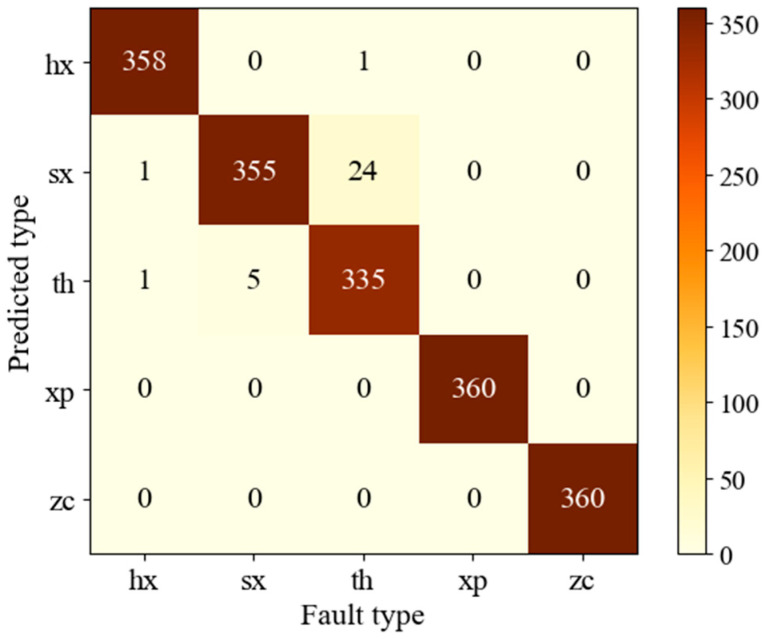
The confusion matrix of the testing samples in the fifth trial.

As one of the visualization tools in artificial intelligence, a confusion matrix is employed for precision evaluation of classification, especially in the process of supervised learning. In order to analyze and discuss the misclassification of the model, a confusion matrix was used for simply and intuitively presenting the statistical classification and misclassification result of each fault type. The proposed model showed a favorable diagnosis accuracy for the non-linear and non-stationary signals. The accuracy reached 100% in the condition of xp and zc (Figure 10). The misclassification was primarily concentrated in the conditions of th and sx, 24 samples in the condition of th are misclassified into sx and one sample is misclassified into hx. The potential reasons could be that the hidden features in the image of sx and hx are similar for CNN, and it is hard to distinguish some of the learned information.

In allusion to the complicated and unintelligible internal operations of CNN, it is of great significance to uncover the mysterious mask to reveal the potential automatic learning process. The visualization of feature learning results was conducted to demonstrate the performance of the model.

The feature extraction of major layers were selected to observe the effectiveness of the model, involving five convolutional layers (Conv 1, Conv 2, Conv 3, Conv 4, Conv 5) and three fully connected layers (FC 1, FC 2, FC 3). Meanwhile, the results of raw input data are taken for comparisons. As a powerful nonlinear dimension reduction algorithm, t-SNE is employed to reduce the high-dimensional feature representations to two dimensions [57].

**Figure 11:**
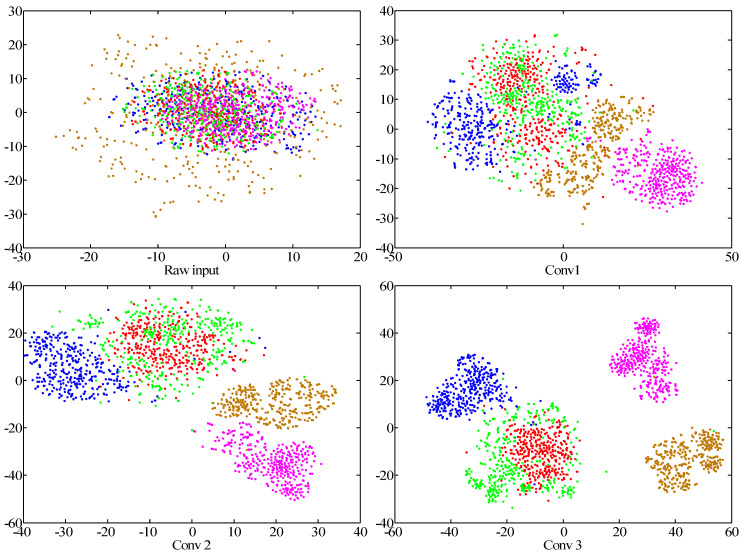

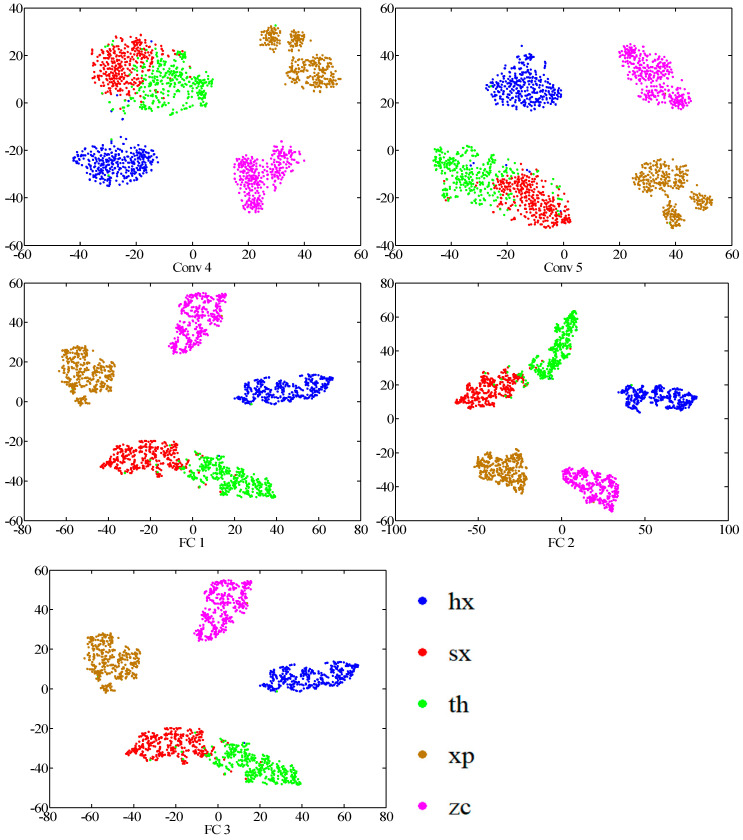
Visualization of different layers via t-SNE: feature representations for the raw input, five convolutional layers and the last fully connected layer, respectively.

The visualization results represent the first two dimensions of the features obtained from t-SNE. Each point denotes a testing sample. The horizontal and vertical axes display the dimensions of t-SNE. It is worth pointing out that the values of each axis express the results after dimension reduction via t-SNE. It can be found that the useful features of the testing datasets are effectively extracted and represented. From early Conv layers to the final FC layers, the features of different fault categories present an increasingly clear classification, as can be seen from Figure 11.

In consideration of raw input, the distributions of the five fault types are almost uniform, which indicates that it is hard to identify the specific types at this stage. After convolutional operation, the features of some fault types start to cluster together. As a whole, overlay phenomena of fault features in the previous layers are apparent. Especially, there is an obvious overlap of various features in the first two layers. As shown in Conv 1, the features of most fault types are scattered points, only the features in the condition of zc present clear clustering; moreover, serious overlays are observed in the features of the four fault types. The features of the other two conditions begin to cluster in Conv 2, xp and hx, respectively; nevertheless, the representations of sx and th are mixed with each other and it is hard to distinguish either of the two types. In view of the FC layers, some crossover areas can be found in the condition of both sx and th, which indicates that misclassification between the two types of faults may occur with this method. However, the feature representations of different fault types become very discriminative, and the features of the same fault types are clustered into the same region. It can be indicated that the low-hierarchical features are converted into high-hierarchical ones through different network layers and the fault classification performance can be enhanced.

### 4.4. Contrastive Analysis

In order to further explore the diagnostic performance of the proposed CNN, different CNN models were employed for comparisons, including Traditional LeNet 5 (T-LeNet 5), Improved LeNet 5 (I-LeNet 5), the CNN containing three convolutional layers (CNN-3), the CNN containing four convolutional layers (CNN-4), and Traditional AlexNet (T-AlexNet).

**Figure 12:**
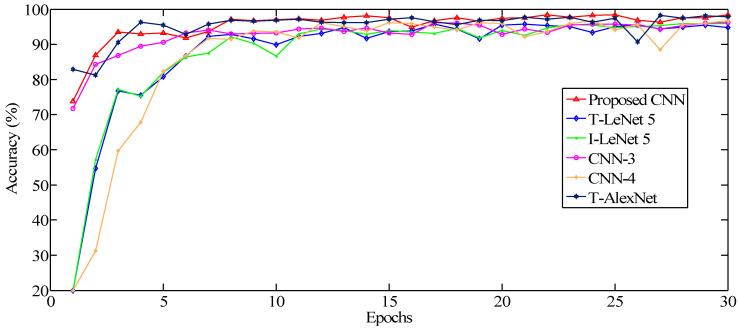
The curve of testing accuracy for different CNN models.

From Figure 12, it can be seen that the convergence effect of the proposed CNN is better than that of other models. During the early stages of feature learning, the CNN models based on LeNet 5 present a lower accuracy. When it reaches over 10 epochs, the accuracy of the proposed CNN is more than 96%, but it is lower than 90% for the LeNet 5 based diagnostic method.

As can be seen from Table 3, the average accuracy of the proposed CNN reached 98.44% and the lower standard deviation (STD) was only 0.001171. The classification accuracy of Traditional LeNet 5 was only 95.22%, which was obviously inferior to the proposed model and to the other models. The proposed model outperformed the other models, indicating a higher average accuracy and a lower STD. It can be implied that the proposed CNN displayed good classification performance and stability for hydraulic pump faults.

**Figure 13:**
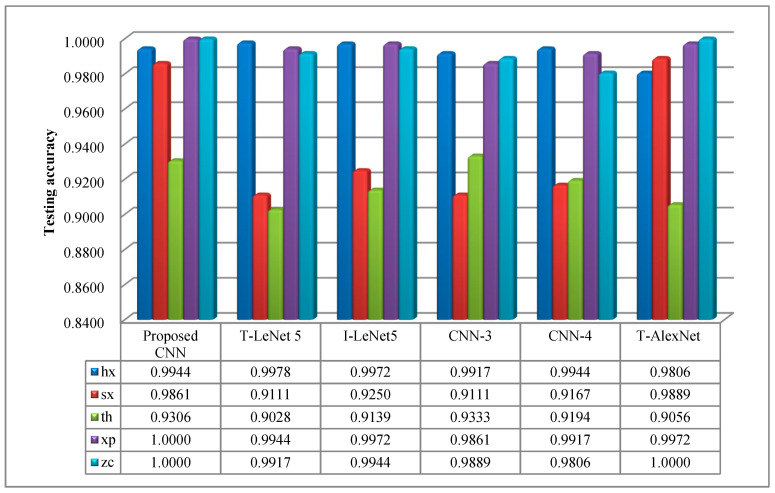
The curve of testing accuracy for different CNN models.

For the purpose of observing the classification effectiveness of each different fault type, respectively, the same models were used for contrastive analysis. From Figure 13, it can be seen that no obvious difference was obtained considering the classification effect on the three conditions, including zc, xp and hx. However, as for the conditions of sx and th, the proposed CNN model was a slightly superior to the other CNN models. The distinction of the two types of faults will be considered as the emphasis of following research.

## 5. Conclusions

In this paper, an integrated deep learning method was constructed on the basis of CNN for fault diagnosis in a hydraulic axial piston pump. The diagnostic performance was validated by the experiments on the hydraulic pump testing platform.

In consideration of the deficiencies in directly using raw vibration signals for feature extraction, CWT was employed to convert time series signals into time-frequency images. The converted images could provide more useful feature information to be used for the deep model.

In light of the remarkable superiority in image classification, CNN is established for feature extraction and fault classification. Adam is used for parameter optimization of the model. Moreover, the dropout strategy is designed in the fully connected layers.

The effectiveness and feasibility of the proposed method is demonstrated by the fault experiment. The faults of the hydraulic pump test rig include hx, sx, xp, th and zc. The highest accuracy of 100% can be achieved in the health condition of zc and xp. The average accuracy can reach up to 98.44%, which is superior to that of other CNN models. The stability of the model is demonstrated by the results of the repeated trials. Furthermore, the effectiveness of the model is demonstrated by t-SNE, and the features after dimension reduction represent the learning consequence of CNN. It can be indicated that the proposed model presents the desirable visualized classification performance for different fault types in a hydraulic axial piston pump. Therefore, the proposed model can automatically learn the useful fault features from a visually similar time-frequency representation. The proposed CNN model effectively overcomes the exiting shortcomings of conventional methods in terms of complex feature extraction and severe dependence on diagnostic knowledge and experience.

Although the model is not desirable for the fault type of th, the classification performance of the model presents an advantage compared with other methods. In future research, different search algorithms will be exploited for the optimization of the model, such as random search and grid search. In addition, enhancement of the input data will be taken into account. The conversion from raw signals to images will be accomplished through other data preprocessing methods for promoting the performance of the network model.

## Figures and Tables

**Table 1:** The health conditions and type labels of hydraulic axial piston pump.

Health Condition	Description	Index Names	Type Labels
Normal	no fault in hydraulic pump	zc	0
Faulty	swash plate wear	xp	1
	loose slipper failure	sx	2
	slipper wear	hx	3
	central spring wear	th	4

**Table 2:** The number and label configuration of datasets for hydraulic axial piston pump under 5 conditions.

Fault Type	Fault Description	Time-Frequency Image	Train Dataset	Test Dataset	Type Labels
hx	slipper wear	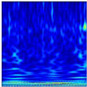	840	360	0
sx	loose slipper failure	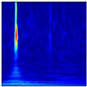	840	360	1
th	central spring wear	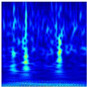	840	360	2
xp	swash plate wear	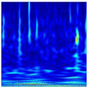	840	360	3
zc	no fault in hydraulic pump	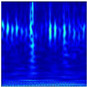	840	360	4
total	-	-	4200	1800	-

**Table 3:** The number and label configuration of datasets for hydraulic axial piston pump under 5 conditions.

CNN Models	Average Accuracy (%)	STD
T-LeNet 5	95.22	0.007472
I-LeNet 5	96.36	0.001172
CNN-3	96.70	0.004603
CNN-4	96.20	0.008946
T-AlexNet	95.87	0.003608
Proposed CNN	98.44	0.001171
